# Severe acute kidney injury related to haemolysis after pulsed field ablation for atrial fibrillation

**DOI:** 10.1093/europace/euad371

**Published:** 2024-01-04

**Authors:** Sandrine Venier, Nathan Vaxelaire, Peggy Jacon, Adrien Carabelli, Antoine Desbiolles, Frederic Garban, Pascal Defaye

**Affiliations:** 1 Department of Cardiology, Electrophysiology Unit, University Hospital of Grenoble Alpes France, CS10217, 38043 Grenoble Cedex 9, France; 1 Department of Cardiology, Electrophysiology Unit, University Hospital of Grenoble Alpes France, CS10217, 38043 Grenoble Cedex 9, France; 1 Department of Cardiology, Electrophysiology Unit, University Hospital of Grenoble Alpes France, CS10217, 38043 Grenoble Cedex 9, France; 1 Department of Cardiology, Electrophysiology Unit, University Hospital of Grenoble Alpes France, CS10217, 38043 Grenoble Cedex 9, France; 1 Department of Cardiology, Electrophysiology Unit, University Hospital of Grenoble Alpes France, CS10217, 38043 Grenoble Cedex 9, France; Department of Hematology, University Hospital of Grenoble Alpes France, CS10217, 38043 Grenoble Cedex 9, France; 1 Department of Cardiology, Electrophysiology Unit, University Hospital of Grenoble Alpes France, CS10217, 38043 Grenoble Cedex 9, France

**Keywords:** Atrial fibrillation, Pulsed field ablation, Haemolysis, Acute kidney injury

## Abstract

**Aims:**

Pulsed field ablation (PFA) has been proposed as a novel alternative to radiofrequency (RF) and cryoablation in the treatment of atrial fibrillation (AF). Following the occurrence of two cases of acute kidney injury (AKI) secondary to haemolysis after a PFA procedure, we evaluated haemolysis in a cohort of consecutive patients.

**Methods and results:**

Two cases of AKI occurred in last May and June 2023. AKI was secondary to acute and severe haemolysis after a PFA procedure. From June 2023, a total of 68 consecutive patients (64.3 ± 10.5 years) undergoing AF ablation with PFA were enrolled in the study. All patients had a blood sample the day after the procedure for the assessment of haemolysis indicators. The pentaspline PFA catheter was used with a total number of median applications of 64 (54; 76). Nineteen patients (28%) showed significantly depleted haptoglobin levels (<0.04 g/L). A significant inverse correlation was found between the plasma level of haptoglobin and the total number of applications. Two groups were compared: the haemolysis+ group (haptoglobin < 0.04 g/L) vs. the haemolysis− group. The total number of applications was significantly higher in the haemolysis+ group vs the haemolysis - group respectively 75 (62; 127) vs 62 (54; 71) *P* = 0.011. More than 70 applications seem to have better sensitivity and specificity to predict haemolysis.

**Conclusion:**

Intravascular haemolysis can occur after certain procedures of PFA. Acute kidney injury is a phenomenon that appears to be very rare after a PFA procedure. However, caution must be exercised in the number of applications to avoid severe haemolysis.

## Graphical Abstract

**Graphical Abstract:**
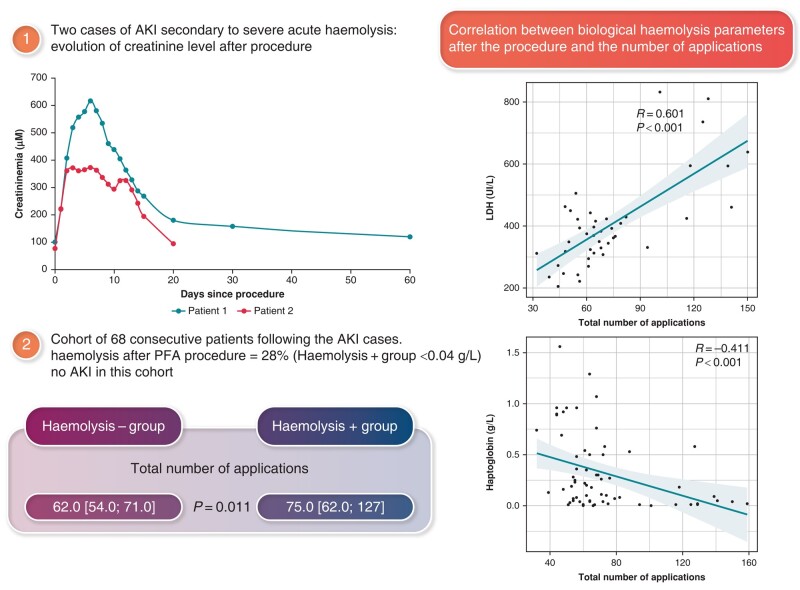

**What’s new?**

- Acute haemolysis may occur in patients who have undergone pulsed field ablation (PFA) with a pentaspline catheter.
- There is an inverse correlation between plasma haptoglobin levels and the total number of PFA applications.
- An average of 70 applications per procedure seems to be reasonnable in routine practice to avoid haemolysis complications.
- Acute kidney injury as a result of haemolysis is still very rare and has occurred only in cases where the total number of applications has exceeded 100.

## Introduction

In recent years, pulsed field ablation (PFA) has been proposed as a novel alternative to radiofrequency (RF) and cryoenergy-based ablation to achieve pulmonary vein isolation (PVI) in the management of atrial fibrillation (AF).^1^ Pulsed field ablation is a non-thermal ablative approach in which cardiomyocyte death is obtained through irreversible electroporation (IRE), while theoretically sparing collateral tissues or red blood cells (RBCs).^2^ Recent studies have shown that PFA using a pentaspline catheter could safely and effectively treat patients with persistent AF, achieving not only PVI but also left atrial posterior wall (LAPW) and mitral isthmus (MI) ablation.^3–5^ Very few complications related to the PFA procedure have been reported, such as phrenic nerve paresis and coronary artery spasm.^4^ To the best of our knowledge, haemolysis after PFA has never been published.

In the past 12 months, our high-volume centre has performed >350 ablations for both paroxysmal and persistent AF using PFA, with favourable results in terms of efficacy and safety. The number of pulse field applications for PVI is contingent upon factors such as vein anatomy and left atrial size and may range from 32 to 60. Additionally, for adjunctive ablations on LAPW and MI, the requirement often exceeds 40 and can go beyond 100.

In May 2023, two patients presented with severe renal failure after extensive use of PFA. Since then, patients have been systematically screened for haemolysis on the first day of treatment.

The aim of our study is to describe the two cases of renal failure that occurred in May and June 2023 and then to describe the factors favouring the occurrence of haemolysis after a PFA procedure in a cohort of 68 patients.

## Methods

In two patients, renal injury due to intravascular haemolysis (IH) after a PFA procedure occurred in May and June 2023. First of all, a clinical description of these two patients was made. From then on, consecutive patients with the PFA procedure were prospectively enrolled in a case series study from June to October 2023. Biological tests were performed between 3 and 5 days prior to the PFA procedure. The parameter characteristics of haemolysis were analysed from blood samples taken at Day 1 after the procedure. The haemolysis markers such as lactate dehydrogenase (LDH), haptoglobin, bilirubin, platelet count, and free plasma haemoglobin were evaluated. In terms of diagnostic sensitivity, haptoglobin and to a lesser degree unspecific LDH were considered superior to the other haemolysis parameters. Then, two groups were compared for the presence of a predictor of haemolysis. One group with significantly diminished haptoglobin levels after the procedure (haemolysis+ group) was compared with a group with normal levels of haptoglobin (haemolysis− group).

### Procedure

All procedures were performed using a strict protocol of conscious sedation and analgesia with a continuous infusion of propofol and bolus of nalbuphine administered by an anaesthesiologist. No patient was intubated, and no muscle relaxant was used. Oral anticoagulant was continued on the day of the procedure.

Two femoral echo-guided venous punctures were performed to obtain venous access. A decapolar catheter was positioned in the coronary sinus. The transseptal puncture was performed with an SL0 sheath. After positioning a guidewire in the left superior pulmonary vein (PV), the SL0 sheath was exchanged for a Faradrive sheath (Faradrive, Boston Scientific Inc., Marlborough, MA, USA), and a 12 Fr over-the-wire PFA ablation catheter (Farawave, Boston Scientific Inc.) was advanced into the left atrium. Heparin (100 IU/kg) was administered immediately after the transseptal puncture, with a target-activated clotting time of between 250 and 300 s.

The PFA system and methods are described in previous publications.^1^ The PFA catheter had five splines each containing four electrodes and could be deployed in either a ‘flower petal’ or a ‘basket’ configuration. When fully deployed in the flower form, the maximum diameter of the distal portion was 31 or 35 mm, depending on the catheter size. The catheter was advanced over a guidewire until the splines achieved circumferential contact/proximity with the PV antra. To ensure contact between the catheter and the PV ostium/antrum, we used fluoroscopy. To achieve contact in the basket configuration, the catheter should not be able to advance any further. To achieve contact in the flower configuration, the petals should be flattened or slightly twisted backwards. No contrast was required.

All PVs were isolated. The number of pulsed field applications for PVI was contingent upon factors such as vein anatomy and left atrial size and may range from 32 to 60. Additionally, for adjunctive ablations of LAPW or MI, often over 40 applications were required and could exceed 100. For LAPW ablation, the catheter was placed in the flower configuration and positioned along the LAPW to deliver overlapping sets of pulses at each location.

### Biological analyses

Each patient had the usual pre-operative blood tests with full blood count, coagulation, and brain natriuretic peptide. A second blood sample was taken the next morning after the procedure for the assessment of the following haemolysis indicators: haptoglobin, free haemoglobin, indirect bilirubin (total bilirubin and direct bilirubin), LDH, and reticulocyte count (Rt). The indicators of the haemolysis mechanism were obtained via a peripheral blood smear, a direct antiglobulin test, and a schizocyte count. Haemoglobinuria was also assessed on Day 1. The principle of the haemoglobinuria test is based on the peroxidase activity of haemoglobin, which catalyses the reaction of diisopropylbenzene dihydroperoxide and 3,3′,5,5′-tetramethylbenzidine. If a uniform colouration appears (from yellow to green-blue +/− dark), haemoglobin or haemolysed RBCs are detected. The results are then reported as +/−, +, ++, or +++. As the most sensitive test for haemolysis, the haptoglobin test was chosen to compare the two groups.

### Statistical analysis

Continuous variables are reported as mean and standard deviation (SD) or median and inter-quartile range (25th and 75th percentiles) according to their Gaussian distribution. Categorical variables are expressed as absolute frequencies and percentages. In the comparative analysis, parametric tests were used for those continuous variables that met the application conditions (e.g. *t*-test) and non-parametric tests (e.g. Pearson *χ*^2^, Fisher, and Wilcoxon) for the ordinal or categorical variables not meeting the parametric criteria. The multivariate analysis model was implemented using the forwards method, including only those variables showing a *P* < 0.15 in the univariate analyses. The results are expressed as odds ratio with 95% confidence intervals. A *P*-value <0.05 was accepted as statistically significant. Pearson’s or Spearman’s correlation coefficients were calculated to test the association between variables (IBM SPSS Statistics, version 24.0, Armonk, NY, USA).

To determine independent predictors of haemolysis, a multivariate logistic regression analysis was performed using depleted haptoglobin (<0.04) as the dependent variable. To find the best model, variables were entered in a forward logistic regression model using Akaike information criteria to determine the best model. Analyses were performed using R software, and *P*  *<* 0.05 was considered statistically significant.

Predictive analysis relied on logistic regression to obtain a probability for each individual to belong to the group haemolysis+. We then drew a receiver operating characteristic (ROC) curve to present the different decision thresholds. The predictive value of the number of applications associated with haemolysis was assessed using an ROC analysis to identify the cut-off point value that maximized sensitivity and specificity.

## Results

### Reports of acute renal injury after a pulsed field ablation procedure

#### Patient 1

A 75-year-old man presented with a recurrence of symptomatic, persistent AF. He had previously undergone two AF ablations with thermal energy. He was referred for PFA. The pentaspline catheter of 31 mm was used. Pulmonary vein isolation, LAPW, and MI ablations were performed. A total of 174 applications were delivered leading to a restoration of sinus rhythm. A few hours later, his urine colour changed to dark brown. His laboratory analyses indicated acute kidney injury, anaemia, and haemolysis. Six hours after the procedure, blood tests revealed a decreased haptoglobin level (0.03 g/L, reference 0.4–2.8 g/L) and high creatinine, bilirubin, and LDH levels (220, 84 µmol/L, and 810 U/L, respectively). His reticulocyte count was normal (49 g/L), and there were no schizocytes. In the urinary analysis, haemoglobinuria was found. A renal ultrasound showed the absence of obstruction in the urinary tract. The patients’ creatinine levels continued to increase until 6 days after the procedure reaching 617 µM and then the levels returned to baseline over a period of 2 months.

#### Patient 2

A 74-year-old man with a history of heart failure with preserved ejection fraction and persistent AF. He had undergone three previous ablation procedures using RF and cryoablation. Due to a recurrence of symptomatic AF, a new procedure was programmed using PFA. His pre-procedural creatininemia was measured at 76 µmol/L (clearance 65 mL/min/m^2^). The procedure consisted of the same sequence as the first patient with 126 applications using the pentaspline 31 mm catheter. The following day, the patient presented biological renal failure with a creatininemia measured at 372 µmol/L and uraemia at 22 mmol/L. No clinical haematuria was observed in this case. As for the first patient, the results of the physical examination and his vital signs were normal. A total of twenty days was necessary for a return to normal creatininemia levels. Haptoglobin was not tested in this patient, because a sample was not systematically collected at that time.

**Figure 1:**
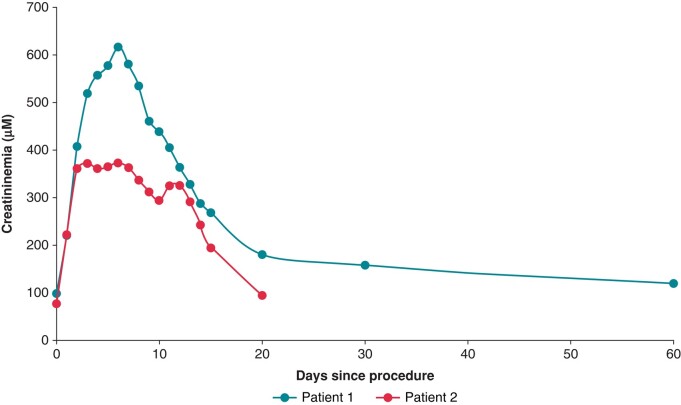
Plasma creatinine levels before and after a PFA procedure for Patients 1 and 2. PFA, pulsed field ablation.

The two patients had maintained preserved diuresis. *Figure 1* summarizes the progression of renal function in the days following the procedure for both Patients 1 and 2.

### The cohort of 68 consecutive patients

A total of 68 consecutive patients (64.3 ± 10.5 years) were studied from June to October 2023. One patient had chronic renal failure with a high creatinine level prior to the procedure. No acute post-procedure renal injury was observed in the following 68 patients enrolled in the study. Baseline characteristics of 68 patients are detailed in *Table 1*. Procedural characteristics are given in *Table 2*.

**Table 1:** Baseline demographic, clinical, electrocardiographic, and AF subtype of the study population

Baseline patient characteristics	*N* = 68 patients
Demographics	
Age (years)	64.3 (±10.5)
Male (%)	75%
Body mass index (kg/m^2^)	27.8 (±5.7)
CHA_2_DS_2_-VASc score	1.9 (±1.4)
Echocardiography parameters	
LA size (mm)	45.5 (± 9.3)
LVEF, *n* (%)	58.9 (7.7)
Past medical history	
Hypertension, *n* (%)	30 (44)
Diabetes, *n* (%)	8 (12)
Dyslipidaemia, *n* (%)	8 (12)
Congestive heart failure, *n* (%)	14 (21)
Coronary artery disease, *n* (%)	6 (9)
Stroke/TIA, *n* (%)	6 (9)
Sleep apnoea, *n* (%)	8 (12)
Chronic renal failure, *n* (%)	1 (1.4)
Chronic obstructive pulmonary disease, *n* (%)	1 (1.4)
Medications	
Vitamin K antagonist, *n* (%)	2 (3)
DOAC, *n* (%)	66 (97)
Anti-arrhythmic drug use, *n* (%)	38 (56)
Indication for ablation	
Paroxysmal atrial fibrillation, *n* (%)	24 (35)
Persistent atrial fibrillation, *n* (%)	20 (29)
Long-standing persistent AF, *n* (%)	24 (35)

Values are expressed as mean (SD) or percentage.

LA size, left atrial size; LVEF, left ventricular ejection fraction; TIA, transient ischaemic attack; DOAC, direct oral anticoagulant; AF, atrial fibrillation.

**Table 2:** Procedural characteristics of the study population

Procedural characteristics	*N* = 68
Procedure duration (min)	54.0 (40.0; 61.5)
LA time (min)	23 (19; 31)
Fluoroscopy time (min)	11.9 (8.5; 14.5)
Fluoroscopy dose (cGy cm^2^)	1276 (910; 2024)
Total number of applications	64 (54; 76)
PVI applications only	42 (61%)
Total basket configuration applications	21 (18; 28)
Total flower configuration applications	34 (28; 38)
PVI + additional non-pulmonary vein ablation	26 (38%)
Catheter size 31 mm	67 (98%)

Values are expressed as mean (SD) or median (quartiles: 25; 75). The total basket or flower configuration applications indicate the number of applications in the flower or basket configuration per PVI procedure.

LA time, left atrial time; PVI, pulmonary vein isolation.

### Biological data on the first day after the procedure

Among 68 patients tested on Day 1 of the procedure, 19 (28%) showed significantly depleted haptoglobin levels (below 0.04 g/L), 27% exhibited haemoglobinuria, LDH levels were elevated (>246 UI/L) in 77% of patients, and plasma-free haemoglobin was elevated (>0.05 g/L) in 35% of patients. Fifty-one patients (72%) had an elevated bilirubin level (>19 μmol/L), and 19% of patients were positive for haemoglobinuria. Only the two patients described above had a significant increase in creatinine levels after the procedure. In the cohort, the median creatinine level was not significantly increased after the procedure. The 19 patients who had haemoglobinuria had low haptoglobinaemia. No schizocytes were found in any of the samples. Biological characteristics before and after the procedure are given in *Table 3*.

**Table 3:** Comparison of baseline biological characteristics and haptoglobin 3–5 days before the procedure and 1 day after the procedure

Biological characteristics	Before procedure	After procedure (Day 1)	*P*-value
Haemoglobin (g/dL)	14.5 (13.7; 15.4)	12.9 (12.1; 14.2)	0.0001
Platelets (10^9^/L)	235 ± 58	193 ± 44	0.001
Leucocytes (10^9^/L)	6.3 (5.2; 6.9)	8.4 (7.4; 9.7)	0.0001
Haptoglobin (g/L)	1.40 (1.20; 1.58)	0.18 (0.04; 0.51)	0.0001
GFR (mL/min/1.73 m^2^)	72 ± 20	80 ± 50	0.96
Creatininemia (μm/L)	73 (73; 104)	80 (71; 102)	0.32
Urea (mmol/L)	7.0 (5.6; 8.2)	6.2 (5.4; 7.5)	0.14
Free haemoglobin (g/L)	/	0.04 (0.02; 0.08)	
LDH (UI/L)	/	374 (311; 445)	
Haemoglobinuria	/	19 (28%)	
Total bilirubin (μm/L)	/	24.5 (18.0; 36.0)	
Direct bilirubin (μm/L)	/	10 (8; 12)	

All biological markers of haemolysis were not sampled before the procedure. Values are expressed as mean (SD) or median (quartiles: 25; 75).

GFR, glomerular filtration rate LDH, lactate dehydrogenase.

**Figure 2:**
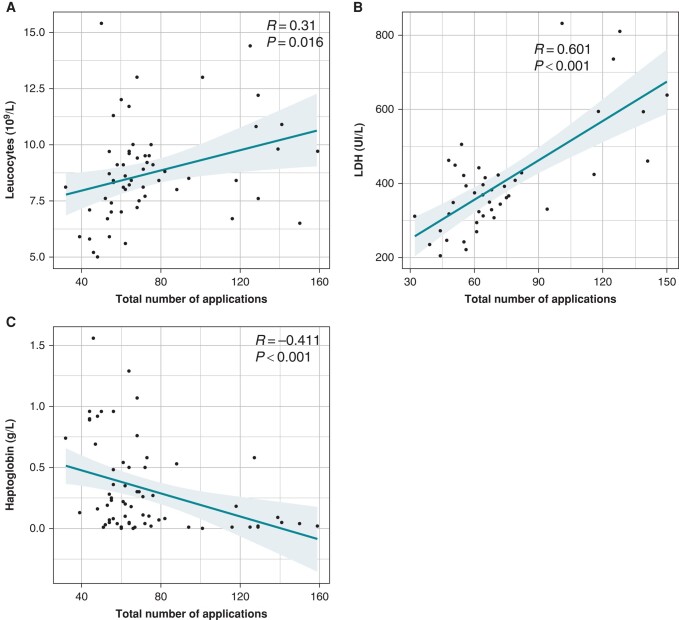
Correlation between biological parameters and the number of applications. (*A*) Leucocytes and the number of applications (*R* = 0.310, *P* = 0.016). (*B*) LDH and the number of applications (*R* = 0.601, *P* < 0.001). (*C*) Inverse correlation between haptoglobin and the number of applications (*R* = −0.411, *P* < 0.001). LDH, lactate dehydrogenase.

There was a non-significant positive correlation between the total number of applications and the creatinine level after the procedure. There was a significant positive correlation between the total number of applications and the urea level (*Figure 2*).

### Comparison between the haemolysis+ group vs. the haemolysis− group

**Figure 3:**
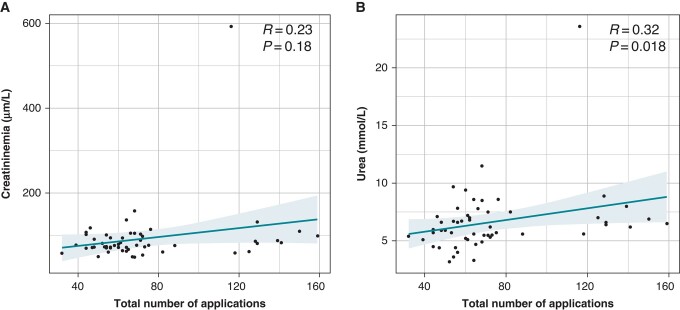
Correlation between the total number of applications and the biological parameters of renal function. (*A*) Correlation between the total number of applications and the creatinine level after ablation (*R* = 0.23, *P* = 0.18). (*B*) Correlation between the total number of applications and the urea level after the procedure (*R* = 0.32, *P* = 0.018).

**Figure 4:**
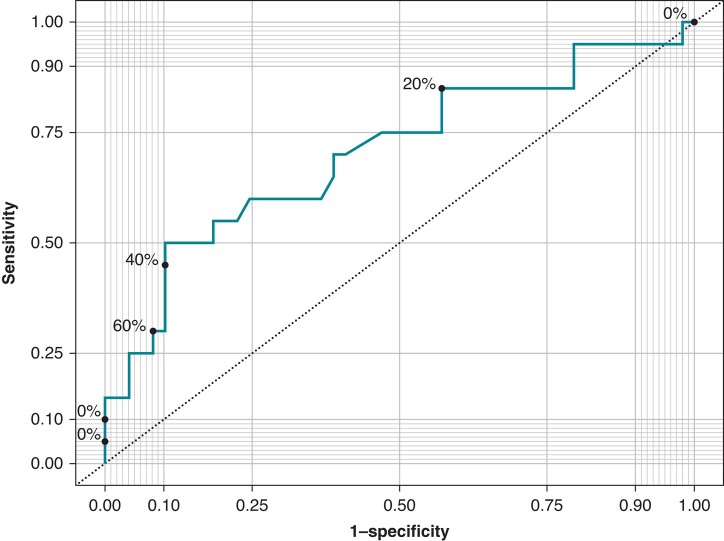
Area under the curve (AUC), sensitivity, specificity, and optimal cut-off values for predicting a collapsed haptoglobin level (<0.04 g/L) on the first day after surgery by the number of applications. The ROC curve shows better sensitivity and specificity at around 70 applications (AUC: 0.709). ROC, receiver operating characteristic.

A comparison of baseline, biological, and procedural characteristics between the haemolysis+ group and the haemolysis− group is presented in *Table 4*. The total number of applications and the left atrial time were significantly higher in the haemolysis+ group in univariate analysis. In multivariate analysis, the total number of applications was significantly higher in the haemolysis+ group (*P* < 0.01). A positive correlation was found among LDH, leucocytes, and the total number of applications (*Figure 3A* and *B*). There was an inverse correlation between the plasma haptoglobin level and the total number of applications (*Figure 3C*). The ROC curve shows better sensitivity and specificity for haemolysis at around 70 applications (*Figure 4*).

**Table 4:** Comparison of baseline demographic, clinical, and procedural characteristics of the haemolysis+ group vs. the haemolysis− group

	Haemolysis− (*n* = 50)	Haemolysis+ (*n* = 18)	*P*-value
Age	66.3 (9.61)	59.2 (11.4)	0.024
Male	37 (74%)	16 (89%)	0.056
BMI	27.5 (24.6; 32.3)	25.3 (24.2; 28.7)	0.17
CHA_2_DS_2_-VASc	2.0 (1.0; 3.0)	1.5 (1.0; 3.0)	0.55
LVEF	60 (60; 65)	60 (60; 65)	0.47
Hypertension	22 (44%)	8 (44%)	0.81
Diabetes mellitus	6 (12%)	2 (11%)	1
Dyslipidaemia	6 (12%)	2 (11%)	1
Congestive heart failure	10 (20%)	3 (17%)	0.74
Coronary artery disease	5 (10%)	3 (17%)	1
Stroke/TIA	3 (6%)	3 (17%)	0.34
Sleep apnoea	6 (12%)	4 (22%)	0.68
Renal failure	1 (2%)	0 (0%)	1
Chronic obstructive pulmonary disease	1 (2%)	0 (0%)	1
BNP	488 (182; 758)	143 (115; 437)	0.28
Haemoglobin (g/dL)	12.9 (13.0)	13.4 (14.2)	0.2
GFR (mL/min/1.73 m^2^)	72.1 (19.0)	70.3 (24.5)	0.81
Creatininemia post-procedure (μm/L)	77 (71; 94)	81 (74; 102)	0.46
Procedural characteristics			
LA time (min)	21.5 (18.0; 28.0)	28.5 (22.8; 39.0)	0.02
Catheter 31 mm	67 (98%)	68 (100%)	1
Total number of applications	62 (54; 71)	75 (62; 127)	0.011
Procedure time (min)	50.0 (40.0; 60.0)	58.5 (53.5; 63.8)	0.25
LA size (mm)	47 (41; 51)	45 (36; 51)	0.82

Univariate analysis.

BMI, body mass index; LVEF, left ventricular ejection fraction; TIA, transient ischaemic attack; BNP, brain natriuretic peptide; GFR, glomerular filtration rate; LA time, left atrial time; PVI, pulmonary vein isolation; LA size, left atrial size.

## Discussion

Our study highlights the occurrence of haemolysis in a significant number of cases following a PFA procedure; 24% of patients exhibited biological signs of haemolysis after PFA, with the number of pulsed field applications being the most significant risk factor. To our knowledge, this is the first reported series of haemolysis after PFA with clinical impact.

Haemolysis is an accelerated RBC destruction with premature removal from the circulation, which usually causes anaemia. IH occurs when RBCs are destroyed in the blood vessel itself. IH results in circulating free haemoglobin. If the haemolysis is mild, the released haemoglobin is bound by circulating haptoglobin. However, with massive haemolysis, the haptoglobin reserve becomes exhausted. Haemoglobin then dissociates into *αβ* dimers (34 kD) that are small enough to be filtered, resulting in haemoglobinuria, haemoglobin cast formation, and haeme uptake by proximal tubular cells. Like myoglobin, these processes can result in acute tubular necrosis and filtration failure.^6^ In our population, only two patients who both underwent >100 applications of PFA developed acute renal failure with dark brown urine as the first symptom, which was secondarily reversible. One of these patients had iron deficiency and the other had chronic haemolysis, which was detected during follow-up. The transient haemolysis in the cohort of patients subsequently studied had no clinical impact. Although there was a slight correlation between the number of applications and the post-operative urea level, there was no significant difference between pre-operative and post-operative renal function in the cohort. Haemoglobin was also significantly reduced after the procedure, but so were platelets, possibly due to haemodilution in the patients. This phenomenon was also observed after ablation procedures with the other techniques (radiofrequency [RF] or cryoablation). The increased leucocytes are evidence of an inflammatory response after ablation, which is also seen with the other energies.^7–9^ However, haemolysis has never been described after RF or cryoablation procedures.^7^ Since the plasma haptoglobin level increases in case of inflammatory syndrome, its decrease after a procedure is only specific for haemolysis. On the other hand, LDH, which increases in inflammatory syndromes, is less specific for haemolysis.

Pulsed field ablation involves the application of ultra-rapid (microseconds to nanoseconds) electrical pulses to generate strong electrical fields causing, among other effects, irreversible nanoscale pore formation and ultimately, cell death.^10^ Myocardial cells have a lower threshold for dielectric cell membrane breakdown resulting in necrosis for the myocardium than surrounding tissues, making it suitable for cardiac ablation. Pre-clinical and clinical studies have demonstrated that by optimizing voltage amplitude, phasic waveforms, and pulse sequences, one can completely avoid damage to peri-cardiac structures such as the oesophagus and the phrenic nerve.^11,12^ However, the electrical field size needed to cause IRE is ∼700 V/cm for the myocardium and 2500 V/cm for RBC.^13^ With each PFA application, electric fields >2500 V/cm can be reached in the immediate vicinity of the electrodes. Minimal haemolysis appears to be possible with each application; massive ablation requiring a large number of applications could proportionally increase the level of haemolysis until a clinical effect is seen. In our study, the use of FARAWAVE outside the veins was off-label, but this is currently a common practice in persistent AF.^3–5^ The PFA FARAWAVE catheter has five splines with distant electrodes and multiple complex electric fields. It is then difficult to apply these findings to the other PFA catheters, which may have different sizes and configurations.

The MANIFEST-PF multi-national survey included a cohort of 1758 consecutive unselected AF patients from all sites performing PFA with the pentaspline catheter in routine clinical practice. In this unselected AF patient population seen in routine practice, PFA was efficacious for PVI and additional non-PVI lesion sets. It should be noted that although the FARAWAVE catheter received a Conformite Europeene (CE) mark only for the treatment of paroxysmal AF. Thirty five per cent of patients treated had persistent AF. Twenty-seven per cent of patients had additional lesions in addition to PVI, including 14% posterior wall isolation, 2% MI applications, and 1% roof line. The FARAWAVE exhibited a safety profile consistent with preferential tissue ablation.^4^ The EUropean Real World Outcomes with Pulsed Field AblatiOn in Patients with Symptomatic AtRIAl Fibrillation registry provided real-world results from seven European high-volume AF ablation centres on the early use of the novel Farapulse PFA technology.^14^ Non-PVI ablations were performed in 14% of patients (posterior wall isolation, LA isthmus ablation, and cavo-tricuspid isthmus ablation). Some complications were described, but no haemolysis was reported. A recent presentation from Reddy and Ekanem^15^ to the AHA with supplementary data from the MANIFEST study showed cases of acute kidney injury requiring dialysis secondary to haemolysis. This finding has not yet been published but was recently presented.

The recent prospective ADVENT study^16^ reported certain rare adverse events in the PFA arm, such as phrenic nerve injury. This finding may suggest that in certain cases, the observed effect of PFA is not entirely limited to cardiomyocytes, but no cases of haemolysis were reported in this randomized trial.

Pulsed field ablation is a new ablation technique that appears promising in terms of safety and efficacy.^17,18^ However, the use of this technique in persistent AF has not been officially validated, and the number of applications required to obtain permanent lesions outside the veins is not clearly defined. The number of applications should be kept to a minimum, with a maximum cut-off of 70 giving the best sensitivity and specificity. Beyond 100 applications, haemolysis seems inevitable. The patient’s previous condition, such as renal function or age, does not appear to be risk factor for haemolysis. Causative factors need to be determined to prevent the occurrence of severe renal failure in fragile patients. We can hypothesize that a concomitant fragility of RBC (an underlying asymptomatic condition) could account for a scenario leading to acute physical haemolysis. Tests such as Eosin-5-Maleimide binding assay, haemoglobin electrophoresis, G6PD/PK levels, and iron assessment should be performed in patients to identify a predisposition to haemolysis.

### Limitations

This was a single-centre study with a small number of patients. However, there is no bias in measurement or selection, nor confusion, to the best of our knowledge. A complete haematologic assessment is needed to investigate RBC weaknesses or predispositions in the affected patients.

## Conclusions

Pulsed field ablation might not be entirely specific to cardiomyocytes, leading to IH in certain cases. The number of applications appears to be a determining factor in the severity of haemolysis. This must remain well below 100 to avoid severe haemolysis. An average of ∼70 applications per patient seems reasonable in routine use. An hydration protocole could be recommended to avoid the risk of kidney failure. Further studies are needed to identify the conditions leading to this side effect, which may lead to new recommendations regarding limiting the number of PFA applications during AF ablation procedures.

## Data Availability

Sharing of the original data will require a data-sharing agreement. Summary data can be provided on reasonable request.
